# Artificial Intelligence–Driven Electrocardiogram Screening for Asymptomatic Left Ventricular Systolic Dysfunction in the General Population

**DOI:** 10.1016/j.jacadv.2026.102660

**Published:** 2026-03-18

**Authors:** Tae-Min Rhee, Sora Kang, Min Sung Lee, Ga In Han, Ah-Hyun Yoo, Jong-Hwan Jang, Yong-Yeon Jo, Jeong Min Son, Joon-myoung Kwon, Su-Yeon Choi, Hak Seung Lee, Heesun Lee

**Affiliations:** a Department of Internal Medicine, Seoul National University College of Medicine, Seoul, Republic of Korea; b Division of Cardiology, Seoul National University Hospital Healthcare System Gangnam Centre, Seoul, Republic of Korea; c Digital Healthcare Institute, Sejong Medical Research Institute, Bucheon, Republic of Korea; d Medical AI Co, Ltd, Seoul, Republic of Korea

**Keywords:** artificial intelligence, electrocardiogram, heart failure, left ventricular systolic dysfunction, prediction model

## Abstract

**Background:**

Asymptomatic left ventricular systolic dysfunction (LVSD) is a well-established precursor of overt heart failure (HF), yet it often remains undiagnosed in the general population. Artificial intelligence–enabled electrocardiogram (ECG) analysis offers a scalable approach for early detection.

**Objectives:**

The purpose of this study was to evaluate the diagnostic performance of an artificial intelligence–enabled ECG model (AiTiALVSD) for identifying asymptomatic LVSD in a large health screening population.

**Methods:**

In this retrospective, single-center study, we evaluated the AiTiALVSD model among 40,713 self-referred adults who underwent a total of 60,711 ECG-transthoracic echocardiography (TTE) pairs between 2011 and 2023. LVSD was defined as a left ventricular ejection fraction ≤40%. Model discrimination was assessed using the area under the receiver-operating characteristic curve (AUROC) and the area under the precision–recall curve (AUPRC), and diagnostic performance metrics were compared with established HF risk scores.

**Results:**

Among 60,711 ECG–TTE pairs, 32 cases (0.054%) met the criteria for LVSD. The AiTiALVSD model demonstrated excellent discrimination (AUROC 0.973; AUPRC 0.328), with a sensitivity of 90.6%, specificity of 99.4%, positive predictive value of 7.7%, and a negative predictive value of 100%. Established HF risk scores, including the MESA (Multi-Ethnic Study of Atherosclerosis) 5-year HF score and Pooled Cohort Equations to Prevent HF score, showed inferior discrimination (AUROC: 0.696 and 0.672, respectively). The MESA score was not designed to detect prevalent LVSD and was calculated without natriuretic peptide data, which may have disadvantaged its performance in this comparison. Simulation analyses suggested that approximately 1,841 ECGs and 13 confirmatory TTEs would be required to detect one case of LVSD.

**Conclusions:**

In a real-world screening population with an extremely low prevalence of LVSD, the AiTiALVSD model demonstrated high diagnostic accuracy, supporting its potential role as a rule-out screening tool for HF prevention. Prospective validation is warranted.

## Central Illustration

**Figure:**
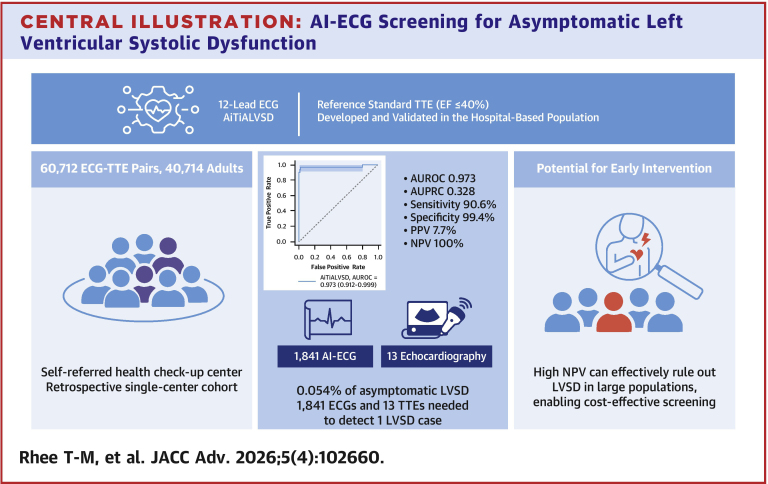

Heart failure (HF) is a major global health burden, affecting over 6 million adults in the United States and more than 55 million individuals worldwide, with substantial impacts on morbidity, mortality, and health care costs.^1^, ^2^, ^3^ Despite advances in HF management, early disease stages often remain undetected in the general population.^4^ Asymptomatic left ventricular systolic dysfunction (LVSD), an early stage of HF characterized by progressive cardiac remodeling, is associated with an over eightfold increased risk of progression to symptomatic HF and a doubling of mortality risk.^5^ Accordingly, recent guidelines recognize LVSD as an early stage of the HF continuum and highlight the need for early identification.^4^^,^^6^ Several clinical risk prediction models have been developed to estimate future HF risk in the general population.^7^^,^^8^ However, these models primarily focus on predicting incident HF rather than identifying existing LVSD.

Electrocardiogram (ECG) is a widely available, noninvasive, and cost-effective tool for cardiovascular assessment. However, conventional 12-lead ECG interpretation has limitations in detecting LVSD, particularly in its early stages.^9^, ^10^, ^11^ With recent breakthroughs in artificial intelligence (AI), there is growing potential to extract subtle patterns from ECG signals, allowing automated and highly accurate LVSD detection.^11^, ^12^, ^13^ Several studies, including our prior work, have demonstrated the strong performance of AI-enabled ECG (AI-ECG) models for LVSD detection using large-scale data sets across diverse clinical settings.^12^^,^^14^, ^15^, ^16^, ^17^, ^18^, ^19^ Nevertheless, many prior studies have focused on hospital-based or ambulatory populations, and evidence from large-scale, self-referred screening settings in the general population remains limited, leaving the role of community-based early screening for LVSD not fully established.^20^^,^^21^

This study aims to externally validate an AI-ECG model for detecting LVSD, originally developed in a hospital-based population, in apparently healthy individuals undergoing ECG as part of a routine health checkup. By leveraging AI-ECG, we seek to assess the prevalence of asymptomatic LVSD in a real-world setting in the general population, extensively validate the AI-ECG for LVSD, and identify individuals at risk who may benefit from further evaluation and intervention. In addition, as a secondary objective, we sought to compare the discriminative performance of the AI-ECG model with established clinical HF risk prediction models. We evaluated this model in a self-referred health checkup cohort in which echocardiography was obtained through participant-selected examination packages.

## Methods

### Study design, setting, and population

**Figure 1:**
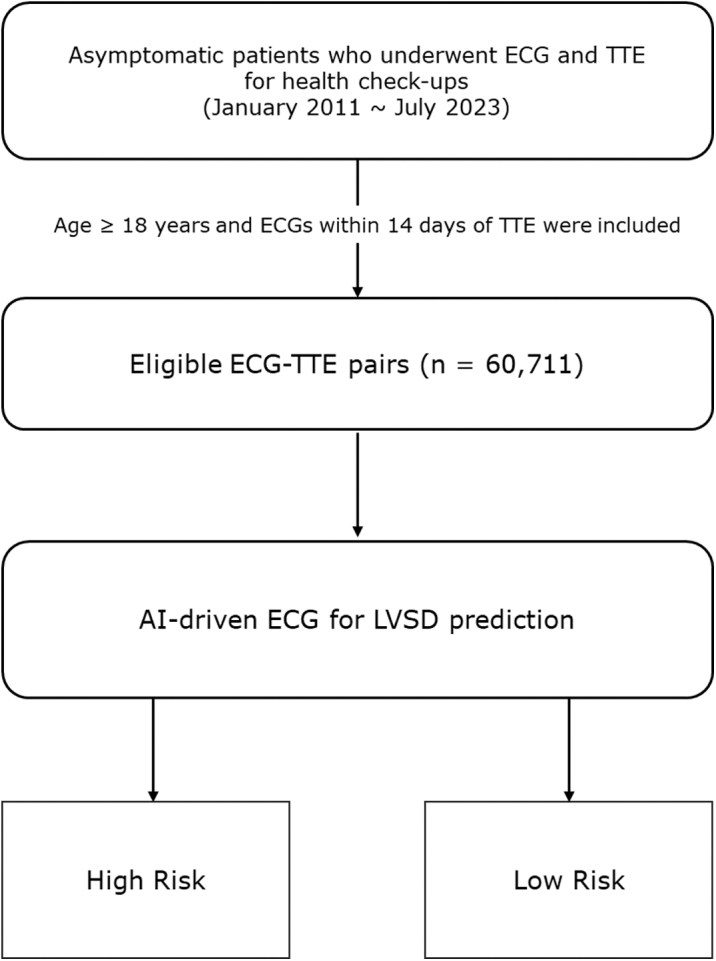
**Study Flow** Between January 2011 and July 2023, a total of TTE and 12-lead ECG pairs were collected from the Seoul National University Hospital Healthcare System. Inclusion criteria were individuals aged 18 years or older who underwent a 12-lead ECG within 14 days of TTE. The final cohort consisted of 60,711 TTE-ECG pairs from 40,713 unique individuals. AI = artificial intelligence; ECG = electrocardiogram; LVSD = left ventricular systolic dysfunction; TTE = transthoracic echocardiography.

This retrospective cohort study was conducted at Seoul National University Hospital Healthcare System Gangnam Center, targeting asymptomatic adults aged 18 years and older who underwent both 12-lead ECG and transthoracic echocardiography (TTE) for the routine health checkup by self-referral between January 3, 2011, and July 31, 2023. In this cohort, TTE was not performed as part of routine clinical screening but was included as part of self-selected health checkup packages chosen by participants. Participants were consecutively enrolled during the study period. Individuals were included if both tests were conducted within a 14-day interval. The primary analysis of this study was conducted at the ECG–TTE encounter level. This approach reflects the real-world self-referred health checkup setting, in which repeated TTE is relatively uncommon. All participants underwent echocardiography as part of their self-selected health checkup programs, rather than based on specific clinical indications. Exclusion criteria included cases with missing or unusable ECG digital files due to noise and those without available left ventricular ejection fraction (LVEF) data in the TTE reports (Figure 1). This study was conducted in accordance with the Declaration of Helsinki, and its protocol was approved by the Institutional Review Board of Seoul National University Hospital (No. H-2307-115-1449). The IRB waived the need for written informed consent due to the retrospective nature of the study, the fully anonymized data set, and the minimal risk to patients.

### Independent variables

Raw 12-lead digital ECG data, recorded for 10 seconds at a 500-Hz sampling rate, were collected and analyzed. During the study period, raw ECG data were recorded using the MAC 2000 ECG device and stored on a MUSE server system. Over time, TTE data were acquired using Vivid 7, E9, or E95 equipment (GE HealthCare Technologies, Inc), with LVEF measured using the modified Simpson’s method. Additional echocardiographic parameters were assessed according to contemporary guidelines relevant to the study period.^22^ Baseline characteristics and medical histories were collected for each individual, including sex, age, height, weight, histories of hypertension, diabetes, dyslipidemia, coronary artery disease, stroke, or smoking history. Laboratory data, including hemoglobin, platelet count, liver function, lipid levels, blood urea nitrogen, creatinine, estimated glomerular filtration rate, glycated hemoglobin, and high-sensitivity C-reactive protein, were also gathered on the same day as the ECG.

### Artificial intelligence electrocardiogram analysis

**Figure 2:**
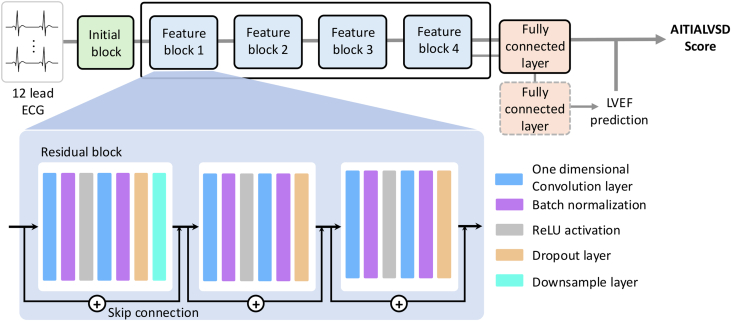
**Architecture of AiTiALVSD Model** ECG = electrocardiogram; LVEF = left ventricular ejection fraction; ReLU = rectified linear unit.

The AI-ECG model used in this study was AiTiALVSD version 1.00.00 (Medical AI Co, Ltd), which was applied without any modification to its algorithm. The development data set did not include any data from this institution, ensuring an independent external validation. The model was originally developed using digital signals derived solely from standard 12-lead ECGs, without incorporating additional clinical variables, and was trained on a large, multi-institutional data set collected from 4 South Korean hospitals. The detailed architecture and development process of the model have been described previously (Figure 2).^16^, ^17^, ^18^, ^19^ Briefly, the model generates a continuous probability score reflecting the risk of LVSD, reported on a scale from 0 to 100. In the present study, this output score was used directly for performance evaluation without retraining or recalibration. Additional methodological details are provided in the Supplemental Methods 1.

### Study outcomes

The primary outcome was the AI-ECG model’s discrimination performance in detecting asymptomatic LVSD, defined as an LVEF of ≤40% on TTE.^6^ The secondary outcome involved comparing the AI-ECG model’s ability to discriminate LVSD with established HF prediction models, specifically the MESA (Multi-Ethnic Study of Atherosclerosis) 5-year HF score and the Pooled Cohort Equations to Prevent HF (PCP-HF) score.^4^^,^^6^ Additionally, exploratory analyses were conducted to evaluate the AI-ECG’s ability to identify cases with an LVEF of ≤50% on TTE.^6^

### Additional analyses

To explore the potential for earlier detection of LVSD, we identified patients labeled as true positives (LVEF ≤40%) who had at least one prior digital ECG recordings available in our database. We retrospectively applied the AiTiALVSD model to these previous ECG recordings and recorded the AI-ECG probability scores. Furthermore, we investigated the echocardiographic characteristics of individuals misclassified as false positives. Specifically, we compared LV size, diastolic function (E/e’), LV hypertrophy, and valvular abnormalities between false positives and the non-LVSD group.

As a sensitivity analysis to ensure strict patient-level independence, all primary performance evaluations were repeated using one index ECG–TTE pair per individual, defined as the most recent eligible pair.

We designed and evaluated a hypothetical diagnostic pathway in which asymptomatic individuals with an abnormal AI-ECG result would undergo a confirmatory transthoracic echocardiogram. The number of AI-ECG tests per detected LVSD case was calculated by dividing the total number of individuals in the target population by the number of identified LVSD cases. Similarly, the required number of TTEs was determined by dividing the number of abnormal AI-ECG findings by the number of true positive confirmations on TTE.

### Statistics

Continuous variables were summarized as medians with IQRs, while categorical variables were presented as counts and percentages. The chi-square test was employed to compare categorical variables between groups, whereas the Mann-Whitney *U* test, a nonparametric method, was applied for continuous variables. In the AiTiALVSD model, a threshold of 9.7 was set to distinguish between low and high risk of LVSD, ensuring a sensitivity of 90%.^16^, ^17^, ^18^, ^19^ The model performance was evaluated using accuracy, area under the receiver-operating characteristic curve (AUROC), AUPRC, sensitivity, specificity, positive predictive value (PPV), and negative predictive value (NPV). Because some individuals contributed more than one ECG–TTE encounter, CIs for all performance metrics were estimated using patient-level clustered bootstrap resampling to account for within-individual correlation arising from repeated measurements.

Threshold CIs were estimated using the bootstrap resampling method. To benchmark the AiTiALVSD model, we compared its performance against conventional HF risk models in the general population.^7^^,^^8^ For the MESA 5-year HF score, NT-proBNP was excluded due to the unavailability of biomarker data, as such test was not part of the routine health checkup program.^7^^,^^8^ The 10-year risk estimation model was derived using the coefficients provided in the PCP-HF.^7^ We additionally evaluated model performance across a range of AiTiALVSD risk score thresholds and examined the impact of threshold selection on sensitivity, specificity, PPV, and NPV. Statistical significance was determined using a 2-sided threshold of *P* < 0.05. All analyses were performed using R software version 4.4.1 and Python version 3.9.7.

## Results

### Baseline characteristics of the study population

Among the total TTE-ECG-matched cases (n = 60,711 from 40,713 individuals), no participants were excluded due to ECG noise or missing data, and 32 (0.054%) were identified as having asymptomatic LVSD, defined as LVEF ≤40% on TTE. When comparing the LVSD group with the non-LVSD group, the median age was 58 years vs 55 years (*P* = 0.082) and the proportion of males was 81.2% vs 64.5% (*P* = 0.372), respectively. The prevalence of coronary artery disease was significantly higher in the LVSD group than in the non-LVSD group (18.8% vs 2.1%; *P* < 0.001). In contrast, the prevalence of other major cardiovascular risk factors—such as obesity, hypertension, diabetes mellitus, dyslipidemia, and smoking history—did not differ significantly between the 2 groups. Echocardiographic evaluation showed that the LVSD group had larger left ventricular chamber sizes in both systole (left ventricular internal dimension in systole) and diastole (left ventricular internal dimension in diastole), a larger left atrial dimension, and higher LV filling pressure (E/e’), accompanied by a significantly lower LVEF (37% vs 66%; *P* < 0.001). Additionally, ECG parameters including heart rate, PR interval, QRS duration, and corrected QT interval were significantly different between the groups. Further details are provided in Table 1.

**Table 1:** Baseline Characteristics of the Study Population

Characteristics	Total (N = 60,711)	LVSD (n = 32)	Non-LVSD (n = 60,679)	*P* Value
Age, y	55.0 (49.0-63.0)	58.0 (51.0-70.0)	55.0 (49.0-63.0)	0.082
Male	39,200 (64.6)	26 (81.2)	39,174 (64.5)	0.420
BMI, kg/m^2^	23.7 (21.8-25.7)	24.0 (21.0-28.0)	23.7 (21.8-25.7)	0.532
Systolic BP, mm Hg	119.0 (110.0-129.0)	124.0 (114.5-127.5)	119.0 (110.0-129.0)	0.542
Diastolic BP, mm Hg	78.0 (70.0-85.0)	74.5 (73.0-86.0)	78.0 (70.0-85.0)	0.917
Hypertension	10,064 (16.6)	6 (18.8)	10,058 (16.6)	0.917
Diabetes mellitus	3,369 (5.5)	3 (9.4)	3,366 (5.5)	0.754
Dyslipidemia	9,120 (15.0)	5 (15.6)	9,115 (15.0)	0.979
Coronary artery disease	1,275 (2.1)	6 (18.8)	1,269 (2.1)	<0.001
Percutaneous coronary intervention	521 (0.9)	4 (12.5)	517 (0.9)	<0.001
Coronary artery bypass graft surgery	93 (0.2)	1 (3.1)	92 (0.2)	<0.001
Stroke	250 (0.4)	0 (0)	250 (0.4)	0.998
Smoking history				0.862
Nonsmoker	14,417 (23.7)	4 (12.5)	14,413 (23.8)	
Ex-smoker	9,092 (15.0)	4 (12.5)	9,088 (15.0)	
Current smoker	7,720 (12.7)	4 (12.5)	7,716 (12.7)	
Echocardiographic parameters				
LVIDd, mm	48.0 (45.0-51.0)	59.0 (55.0-64.0)	48.0 (45.0-51.0)	<0.001
LVIDs, mm	28.0 (25.0-30.0)	47.0 (42.0-50.0)	28.0 (25.0-30.0)	<0.001
LV ejection fraction, %	66.0 (62.0-70.0)	37.0 (34.5-40.0)	66.0 (60.0-73.0)	<0.001
IVSd, mm	9.0 (8.0-10.0)	9.0 (8.0-10.0)	9.0 (8.0-10.0)	0.917
LVPWd, mm	9.0 (8.0-10.0)	10.0 (8.2-10.0)	9.0 (8.0-10.0)	0.093
LA diameter, mm	35.0 (32.0-39.0)	42.0 (38.0-49.0)	35.0 (32.0-39.0)	<0.001
AV diameter, mm	30.0 (28.0-33.0)	32.0 (30.5-34.5)	30.0 (28.0-33.0)	0.004
Mitral inflow E, m/s	0.6 (0.5-0.7)	0.7 (0.5-0.7)	0.6 (0.5-0.7)	0.191
Mitral inflow A, m/s	0.6 (0.5-0.7)	0.6 (0.5-0.8)	0.6 (0.5-0.7)	0.549
Mitral inflow DT, mSec	215.0 (176.0-263.0)	173.5 (137.5-220.0)	215.0 (176.0-263.0)	<0.001
Mitral inflow E/A ratio	0.9 (0.7-1.2)	0.8 (0.6-1.4)	0.9 (0.7-1.2)	0.660
Mitral annulus medial E', cm/s	7.0 (6.0-8.0)	5.0 (4.0-6.0)	7.0 (6.0-8.0)	<0.001
Mitral annulus medial A', cm/s	9.0 (8.0-10.0)	7.0 (5.0-8.0)	9.0 (8.0-10.0)	<0.001
Mitral annulus medial S', cm/s	8.0 (7.0-8.0)	5.0 (4.0-5.6)	8.0 (7.0-8.0)	<0.001
E/E' ratio	8.0 (6.9-10.0)	13.0 (10.0-17.5)	8.0 (6.9-10.0)	<0.001
PASP, mm Hg	28.0 (25.0-31.0)	30.0 (25.5-42.0)	28.0 (25.0-31.0)	0.135
Sinus of Valsalva diameter, mm	39.0 (36.0-42.0)	35.0 (35.0-35.0)	39.0 (36.0-42.0)	0.288
ST junction diameter, mm	32.0 (30.0-35.0)	30.0 (30.0-30.0)	32.0 (30.0-35.0)	0.456
Ascending aorta diameter, mm	37.0 (34.0-39.0)	42.0 (42.0-42.0)	37.0 (30.0-35.0)	0.164
LV mass index, g/m^2^	147.8 (122.3-174.5)	215.7 (174.2-275.0)	147.8 (122.3-174.5)	<0.001
Laboratory findings				
Hemoglobin concentration, g/dL	14.5 (13.5-14.5)	15.4 (14.6-16.2)	14.5 (13.5-15.5)	0.048
Platelet count, 10^3^/μL	229.0 (196.0-266.0)	195.0 (169.5-229.0)	229.0 (196.0-266.0)	0.005
Glucose, mg/dL	99.0 (92.0-107.0)	107.0 (89.5-118.0)	99.0 (92.0-107.0)	0.347
HbA1c, %	5.6 (5.4-5.9)	6.0 (5.7-6.1)	5.6 (5.4-5.9)	0.018
Total protein, mg/dL	7.1 (5.4-5.9)	7.0 (7.0-7.2)	7.1 (6.8-7.4)	0.608
Albumin, mg/dL	4.4 (4.3-4.6)	4.3 (4.2-4.5)	4.4 (4.3-4.6)	0.145
Blood urea nitrogen, mg/dL	14.0 (12.0-17.0)	16.5 (11.5-19.5)	14.0 (12.0-17.0)	0.194
Creatinine, mg/dL	0.9 (0.7-1.0)	1.0 (0.9-1.1)	0.8 (0.7-1.0)	0.005
eGFR, mL/min/1.73 m^2^	85.4 (76.4-95.5)	72.7 (62.6-82.4)	85.4 (76.4-95.5)	0.002
Cholesterol, mg/dL	191.0 (167.0-216.0)	190.5 (164.0-206.5)	191.0 (167.0-216.0)	0.632
Triglyceride, mg/dL	98.0 (69.0-141.0)	118.5 (84.0-165.0)	98.0 (69.0-141.0)	0.142
HDL-cholesterol, mg/dL	52.0 (44.0-61.0)	46.5 (38.0-67.0)	52.0 (44.0-61.0)	0.342
LDL-cholesterol, mg/dL	118.0 (97.0-140.0)	112.5 (78.0-153.5)	118.0 (97.0-140.0)	0.441
AST, mg/dL	23.0 (19.0-28.0)	27.5 (22.0-33.0)	23.0 (19.0-28.0)	0.086
ALT, mg/dL	21.0 (16.0-30.0)	21.5 (16.5-29.5)	21.0 (16.0-30.0)	0.791
GGT, mg/dL	25.0 (17.0-41.0)	39.0 (26.0-78.5)	25.0 (17.0-41.0)	0.026
hs-CRP, mg/dL	0.1 (0.0-0.1)	0.1 (0.0-0.2)	0.1 (0.0-0.1)	0.394
Uric acid, mg/dL	5.5 (4.5-6.5)	6.2 (5.5-7.1)	5.5 (4.5-6.5)	0.054
Electrocardiogram parameters				
HR, beats/min	66.0 (60.0-73.0)	75.5 (63.0-95.5)	66.0 (60.0-73.0)	0.001
PR interval, ms	164.0 (150.0-178.0)	173.0 (161.0-199.0)	164.0 (150.0-178.0)	0.002
QRS duration, ms	92.0 (86.0-100.0)	108.0 (96.0-118.0)	92.0 (86.0-100.0)	<0.001
QT interval, ms	402.0 (384.0-422.0)	412.0 (380.0-430.0)	402.0 (384.0-422.0)	0.583
Corrected QT interval, ms	423.00 (409.0-438.0)	458.5 (438.0-477.5)	423.0 (409.0-438.0)	<0.001
P axis	54.0 (39.0-66.0)	55.0 (39.0-63.0)	54.0 (39.0-66.0)	0.722
R axis	44.0 (17.0-64.0)	24.5 (−8.5-61.0)	44.0 (17.0-64.0)	0.072
T axis	39.0 (24.0-52.0)	47.5 (7.5-85.0)	39.0 (24.0-52.0)	0.398
AiTiALVSD				
Score	0.2 (0.1-0.4)	50.9 (34.0-76.3)	0.2 (0.1-0.4)	<0.001
High risk by AiTiALVSD	377 (0.6)	29 (90.6)	348 (0.6)	<0.001

Values are as n (% or median (IQR). In the AiTiALVSD model, a threshold of 9.7 was used to distinguish between low and high risk of LVSD, which was determined based on the development set to achieve a sensitivity of 90%.

ALT = alanine aminotransferase; AST = aspartate aminotransferase; AV = aortic valve; BMI = body mass index; BP = blood pressure; DT = deceleration time; eGFR = estimated glomerular filtration rate; GGT = gamma-glutamyl transferase; HbA1c = glycated hemoglobin; HDL = high-density lipoprotein; HR = heart rate; hs-CRP = high-sensitivity C-reactive protein; IVSd = interventricular septal thickness in diastole; LA = left atrium; LDL = low-density lipoprotein; LV = left ventricle; LVIDd = left ventricular internal dimension in diastole; LVIDs = left ventricular internal dimension in systole; LVPWd = left ventricular posterior wall thickness in diastole; LVSD = left ventricular systolic dysfunction; PASP = pulmonary artery systolic pressure.

### Study outcomes

**Figure 3:**
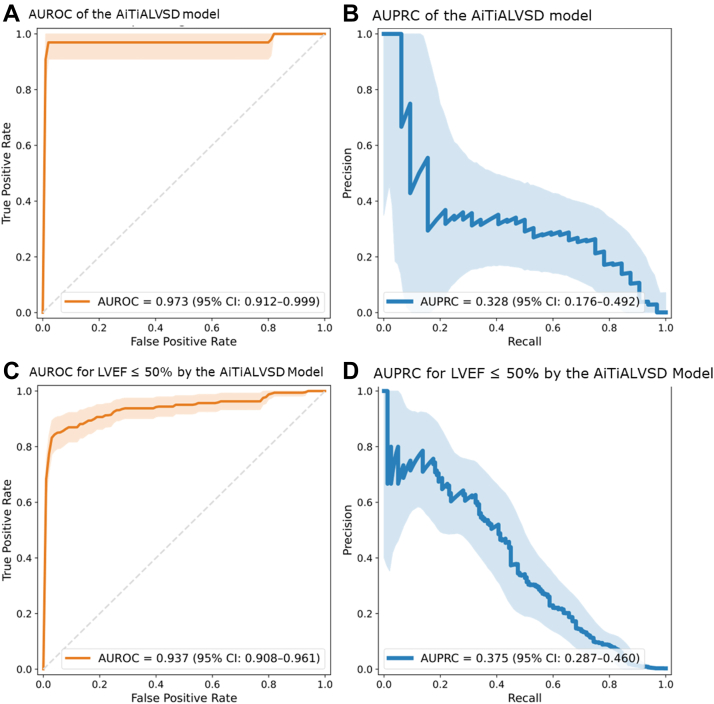
**AiTiALVSD Model Performance in Detecting LVSD** A and C show the ROC curves, indicating the discrimination performance of the AiTiALVSD model in identifying left ventricular systolic dysfunction with 2 thresholds: LVEF ≤40% (AUROC = 0.973) and LVEF ≤50% (AUROC = 0.937). B and D display the corresponding PR curves, with AUPRC values of 0.328 for LVEF ≤40% and 0.375 for LVEF ≤50%. The shaded areas around the curves represent the 95% CIs. AUROC = area under the receiver-operating characteristic curve; AUPRC = area under the precision–recall curve; other abbreviations as in Figures 1 and 2.

Table 2 summarizes the performance metrics of the AiTiALVSD model, and Figures 3A and 3B presents the corresponding AUROC and AUPRC. The AiTiALVSD model demonstrated an AUROC of 0.973 (95% CI: 0.912-0.999) and an AUPRC of 0.328 (95% CI: 0.176-0.492) in detecting asymptomatic LVSD, with a sensitivity of 0.906 (95% CI: 0.789-1.000), specificity of 0.994 (95% CI: 0.994-0.995), PPV of 0.077 (95% CI: 0.050-0.104), and NPV of 1.000 (95% CI: 1.000-1.000). In the final classification, 377 individuals (0.6%) were categorized as high risk by the AiTiALVSD model, while the remaining 60,334 were classified as low risk. Among the 32 patients confirmed LVSD, 29 (90.6%) were correctly classified as high risk. Detailed classification results are provided in Supplemental Table 1. A sensitivity analysis restricted to one index ECG–TTE pair per individual demonstrated similar diagnostic performance, supporting the robustness of the primary encounter-level results (Supplemental Table 2).

**Table 2:** Performance Metrics of the AiTiALVSD for Detecting LVSD

Outcome	AUROC (95% CI)	AUPRC (95% CI)	Sensitivity (95% CI)	Specificity (95% CI)	PPV (95% CI)	NPV (95% CI)
LVEF ≤40%	0.973 (0.912-0.999)	0.328 (0.176-0.492)	0.906 (0.789-1.000)	0.994 (0.994-0.995)	0.077 (0.050-0.104)	1.000 (1.000-1.000)
LVEF ≤50%	0.937 (0.908-0.961)	0.375 (0.287-0.460)	0.586 (0.506-0.662)	0.995 (0.995-0.996)	0.249 (0.204-0.299)	0.999 (0.999-0.999)

AUROC = area under the receiver-operating characteristic curve; AURPC = area under the precision-recall curve; LVEF = left ventricular ejection fraction; NPV = negative predictive value; PPV = positive predictive value; other abbreviations as in Table 1.

**Figure 4:**
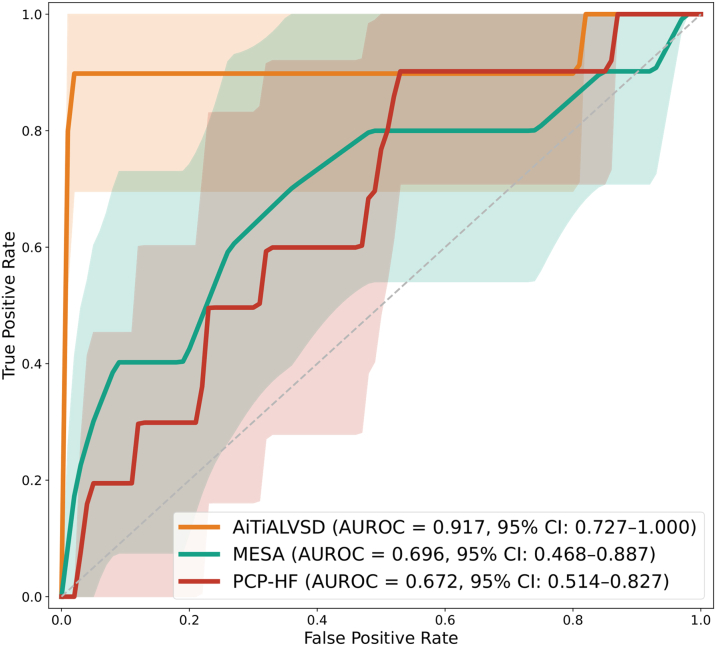
**Comparison of AUROC Curves for AiTiALVSD Model and Conventional HF Prediction Models** AUROC curves illustrate the discrimination performance of the AiTiALVSD model (orange line, AUROC = 0.917) compared to models based on the MESA 5-year HF score (green line, AUROC = 0.696), and the PCP-HF score (red line, AUROC = 0.672). The shaded areas indicate the 95% CIs for each model. MESA = Multi-Ethnic Study of Atherosclerosis; PCP-HF = Pooled Cohort Equations to Prevent HF; other abbreviation as in Figure 3.

For the comparative analysis of the AiTiALVSD model with established HF risk models, individuals with missing clinical variables were excluded, resulting in a final analytic cohort of 21,357. In this secondary analysis, the AiTiALVSD model achieved an AUROC of 0.917 (95% CI: 0.727-1.000), outperforming the MESA 5-year HF score (AUROC: 0.696; 95% CI: 0.468-0.887) and the PCP-HF score (AUROC: 0.672; 95% CI: 0.514-0.827) (Figure 4, Table 3, Supplemental Figure 1). Our simulation results indicated that, to detect one case of LVSD, approximately 1,841 AI-ECG tests and 13 TTEs would be necessary in the overall individuals.

**Table 3:** Comparative Performance Metrics of AiTiALVSD and Other Heart Failure Prediction Models (N = 21,357)

	AUROC (95% CI)	AUPRC (95% CI)	Sensitivity (95% CI)	Specificity (95% CI)	PPV (95% CI)	NPV (95% CI)
AiTiALVSD	0.917 (0.727-1.000)	0.321 (0.083-0.663)	0.807 (0.500-1.000)	0.995 (0.994-0.996)	0.068 (0.026-0.122)	1.000 (1.000-1.000)
MESA 5-y HF	0.696 (0.468-0.887)	0.002 (0.001-0.005)	0.699 (0.375-1.000)	0.640 (0.634-0.646)	0.001 (0.000-0.002)	1.000 (1.000-1.000)
PCP-HF	0.672 (0.514-0.827)	0.001 (0.000-0.002)	0.805 (0.500-1.000)	0.483 (0.476-0.489)	0.001 (0.000-0.001)	1.000 (1.000-1.000)

Risk scores were calculated and compared only among individuals with complete variables for each model. The MESA score excluded NT-proBNP due to unavailable biomarker data. The PCP-HF score was derived using published coefficients.

MESA = Multi-Ethnic Study of Atherosclerosis; PCP-HF = Pooled Cohort Equations to Prevent Heart Failure; other abbreviations as in Tables 1 and 2.

### Exploratory analysis

In this cohort, 160 individuals (0.26%) were identified LVEF ≤50% on TTE. At the LVEF ≤50% threshold, the AiTiALVSD model maintained excellent predictive performance, achieving an AUROC of 0.937 (95% CI: 0.908-0.961) and AUPRC of 0.375 (95% CI: 0.287-0.460), with a sensitivity of 0.586 (95% CI: 0.506-0.662), specificity of 0.995 (95% CI: 0.995-0.996), PPV of 0.249 (95% CI: 0.204-0.299), and NPV of 0.999 (95% CI: 0.999-0.999) (Table 2, Figures 3C and 3D).

### Past ECG analyses in true positives and characteristics of false positives

Among the 32 individuals with LVSD, 16 had at least one prior ECG available in our database (median interval: 70 months before the index ECG). The median AiTiALVSD score on these older ECGs was 6.8 (IQR: 2.0-33.4), and 12 out of these 16 patients exceeded the prespecified threshold (9.7 on a 0-100 probability scale) derived from the development data set to ensure 90% sensitivity. Notably, 12 of these patients showed elevated scores on their older ECGs as well, suggesting that opportunities for earlier detection and intervention may have been missed (Supplemental Figure 2).

A total of 348 cases were classified as false positives, in which LVEF was over 40% on TTE despite being classified as high risk for LVSD by the AiTiALVSD model. A review of their TTEs revealed that only 57 cases (16.4%) were completely normal, while the remaining 291 cases (83.6%) exhibited one or more notable cardiac findings, including coronary artery disease such as previous myocardial infarction (n = 115, 33.0%), atrial fibrillation (n = 71, 20.4%), or valvular heart disease (n = 37, 10.6%).

## Discussion

Our study demonstrates that AiTiALVSD can identify asymptomatic LVSD with strong discriminatory performance in a large, self-referred health checkup cohort characterized by an extremely low disease prevalence (Central Illustration). Despite this low prevalence, the model maintained high sensitivity and rule-out capability, supporting its potential role as a first-line screening tool in population-based settings. In this context, the clinical utility of AI-ECG is best understood not as a definitive diagnostic test, but as a triage tool, with the downstream burden of confirmatory echocardiography being highly dependent on the selected decision threshold.

**Central Illustration:**
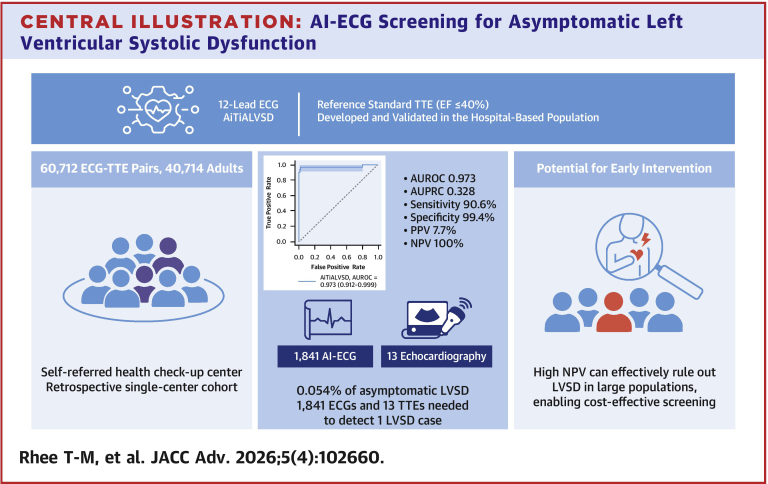
**AI-ECG Screening for Asymptomatic Left Ventricular Systolic Dysfunction** This illustration summarizes the study design, diagnostic performance, and screening efficiency of the AiTiALVSD model in a self-referred health checkup population. A total of 60,711 ECG-TTE pairs from 40,713 adults were analyzed to assess the ability of a 12-lead AI-enhanced ECG (AiTiALVSD) to detect asymptomatic LVSD, defined as LVEF ≤40% on echocardiography. The model demonstrated excellent discrimination (AUROC 0.973, AUPRC 0.328), high sensitivity (90.6%), specificity (99.4%) with a very high negative predictive value in this extremely low-prevalence screening setting. In a cohort with a 0.054% prevalence of asymptomatic LVSD, simulation suggests that 1,841 AI-ECG tests and 13 confirmatory echocardiograms are needed to identify one true positive case. The model’s high NPV allows for efficient rule-out in large populations, supporting its use. EF = ejection fraction; NPV = negative predictive value; PPV = positive predictive value; other abbreviations as in Figures 1 and 2.

### Unique characteristics of a self-referred routine checkup cohort

HF is a chronic condition that worsens over time and is increasing in prevalence.^5^^,^^6^ A meta-analysis estimated that the survival rates for all types of HF are 87% at 1 year, more than half at 5 years, and 35% at 10 years after the onset of clinical symptoms.^23^ Observational data from the Framingham Heart Study indicated that 3.0% of the community had asymptomatic LVSD (LVEF ≤50%), with 1.2% meeting the criterion of LVEF ≤40%.^5^ Similarly, Kashou et al reported a 2.0% prevalence of asymptomatic LVSD (LVEF ≤40%) in a U.S. community-based cohort.^14^ By contrast, our study found a lower prevalence of 0.054%, likely reflecting the unique characteristics of a self-referred health checkup population. Individuals participating in routine examinations may be more proactive about their health or may have had earlier cardiovascular interventions, thereby reducing the proportion who progress to LVSD. Although this selection bias implies that our cohort may not represent the general population, the AI-ECG model’s strong performance in a setting with extremely low LVSD prevalence underscores its potential utility as a screening tool in real-world clinical practice. All echocardiographic evaluations were conducted and interpreted by well-trained cardiologists, further supporting the validity of our findings regarding asymptomatic LVSD in this specialized screening environment.

### Implications for early detection and screening strategies

Timely identification allows for early therapeutic interventions, including guideline-directed medical therapy, which has been proven to increase survival, improve the quality of a patient’s life, and delay HF progression.^4^^,^^6^^,^^24^ Although guidelines recommend echocardiography and BNP measurement alongside clinical assessment for early detection of HF as possible,^6^^,^^25^ routine screening, including TTE in the entire population, is not feasible due to cost, insufficient facilities, and logistical constraints.^6^^,^^25^ Therefore, practical and widely applicable high-sensitivity screening tools are essential for early diagnosis. ECG is a promising candidate for screening in the general population. However, conventional ECG interpretation may not fully realize its potential, as no specific criteria exist for diagnosing LVSD on ECG. With advances in AI, AI-driven ECG analysis has recently emerged as a cost-effective, accurate, and scalable alternative, enabling the identification of at-risk individuals who may require further detailed evaluation.^13^^,^^26^^,^^27^

Given the extremely low prevalence of LVSD in this cohort, the observed high NPV should be interpreted in the context of disease prevalence, as numerically high NPV values may arise even with modest test discrimination. Accordingly, the primary clinical role of AI-ECG in this setting is as a rule-out screening and triage tool rather than a definitive diagnostic test. In this low-prevalence screening setting, decision threshold selection substantially influences PPV and the downstream volume of confirmatory echocardiography (Supplemental Table 3 and 4) and may be tailored to local or institutional screening priorities.

Compared with prior community-based studies, the markedly lower LVSD prevalence observed in this self-referred health checkup cohort highlights the influence of screening context and population characteristics. Nevertheless, the preserved discriminatory performance of AI-ECG in this setting supports its potential utility across diverse screening environments. These findings underscore the resource-intensive nature of screening in extremely low-prevalence populations, emphasizing the importance of careful threshold optimization and follow-up strategies. Accordingly, generalization of these findings should consider differences in health care infrastructure and screening environments.

### Comparative performance and future perspectives

In clinical practice, several HF risk prediction models, such as the MESA 5-year HF score and the PCP-HF score, have been developed for risk stratification and prevention in the general population.^7^^,^^8^ However, these models are primarily designed to predict HF incidence rather than detect asymptomatic LVSD, and they rely on clinical and laboratory parameters that may not always be readily accessible in primary care or health checkup settings. This limitation makes them less ideal for point-of-care screening of LVSD. In contrast, the overwhelming performance of our AI-ECG model—evidenced by its high AUROC—suggests that it can provide additional discrimination beyond traditional clinical parameters. Moreover, in high-volume health screening environments, the intuitive and resource-efficient nature of AI-ECG could minimize the need for extensive clinician involvement, thereby suggesting its potential as a powerful and efficient screening tool.

Our exploratory analysis using an LVEF cutoff of ≤50% further reinforces the robustness of the AiTiALVSD model, with an AUROC of 0.937. Although sensitivity declines at this threshold, the high sensitivity and NPV underscore its utility in safely ruling out disease. Importantly, applying an LVEF cutoff of 50% holds clinical significance because it may indicate the need for regular follow-up and medical evaluation even among individuals without overt symptoms, highlighting the potential of AI-ECG in community-based screening programs.

Notably, the analysis of previous ECGs revealed that many true positive patients had elevated AI-ECG scores months before their formal diagnosis, indicating an opportunity for earlier detection and intervention. Furthermore, among the 348 cases initially classified as false positives, only 16.4% had completely normal TTEs; the remaining cases exhibited structural or functional abnormalities. This observation suggests that some false positives may represent subclinical cardiac pathology that warrants closer clinical evaluation. Together, these findings emphasize that AI-ECG screening not only identifies overt LVSD but could also serve as an early warning system for subclinical dysfunction, potentially optimizing resource use and enhancing patient care in large-scale screening settings.

### Study limitations

Several limitations should be considered when interpreting our findings. First, because this was a single-center, retrospective study of individuals undergoing self-referred health checkups, selection bias cannot be excluded. Such individuals may have fewer comorbidities or be more proactive about their health, which likely contributed to the extremely low prevalence of LVSD (0.054%) observed in our cohort. This unique characteristic may limit the generalizability of our findings to the broader population. Second, the definition of “asymptomatic” relied on the absence of documented HF symptoms rather than structured symptom assessment or formal functional testing, leaving the possibility that some participants may have had unrecognized clinical symptoms. Third, while our findings suggest excellent discrimination by the AiTiALVSD model, the low prevalence of LVSD meant the model’s PPV remained modest; prospective, population-based screening studies in more diverse or higher-risk populations are needed to validate its clinical utility, and additional investigations are warranted to establish its cost-effectiveness. Fourth, we only evaluated LV systolic function using LVEF, and we did not explore subclinical changes such as global longitudinal strain or diastolic dysfunction, which may offer additional insights into early cardiac remodeling. Fifth, we did not assess whether this AI-ECG model's early detection of asymptomatic LVSD would translate into improved clinical outcomes or cost-effectiveness—both critical considerations for widespread screening implementation. Finally, although we compared our model against traditional HF risk scores, future studies incorporating other biomarkers (eg, natriuretic peptides), detailed socioeconomic variables, and outcome data over more extended follow-up periods will be crucial to establish the role of AI-ECG in routine community-based screening. In addition, comparisons with established HF risk scores should be interpreted cautiously, as these models were designed to predict incident HF rather than detect current LVSD. Furthermore, exclusion of N-terminal pro B-type natriuretic peptide due to data unavailability may have disadvantaged the performance of the MESA score in our analysis.

## Conclusions

In this large, single-center health checkup cohort with an extremely low prevalence of asymptomatic LVSD, the AI-ECG model demonstrated excellent diagnostic performance, highlighted by a high AUROC and an excellent NPV. These findings underscore its potential as a scalable screening tool that bridges routine ECGs and resource-intensive echocardiography, outperforming conventional HF risk models. Prospective multicenter trials and cost-effectiveness studies are warranted to confirm its role in broader preventive strategies.

Perspectives **COMPETENCY IN MEDICAL KNOWLEDGE:** AI-ECG can screen for asymptomatic LVSD in routine health checkup populations with extremely low disease prevalence, achieving high discrimination and very high rule-out performance when interpreted in the context of disease prevalence. Compared with conventional HF risk scores, an AI-ECG–first pathway paired with confirmatory TTE for positives can triage large populations efficiently while flagging additional subclinical cardiac abnormalities among apparent “false positives.”

**TRANSLATIONAL OUTLOOK 1:** Prospective, multicenter validation and health-economic analyses are needed to confirm generalizability, quantify downstream testing burden, and determine cost-effectiveness across settings (primary care, employer screening, pharmacies). Implementation work should focus on electronic health record integration, automated reflex testing (TTE or natriuretic peptides), threshold calibration to local priorities, and equity/bias monitoring across demographic subgroups.

**TRANSLATIONAL OUTLOOK 2:** Define standardized care pathways for AI-ECG–positive individuals with normal LVEF (eg, repeat testing, strain imaging, risk-factor optimization) and follow-up intervals to ensure safety.

## Funding support and author disclosures

This study was funded by Medical AI Co, Ltd. Authors affiliated with Medical AI contributed to the study design, data collection, analysis, and interpretation in their capacity as individual researchers. The funder did not impose restrictions on data access, data interpretation, or the decision to submit the manuscript for publication. Drs Kang, Min Sung Lee, Han, Yoo, Jang, Jo, Son, Kwon, and Hak Seung Lee are employees of Medical AI Co, Ltd and hold stocks in the company. All other authors have reported that they have no relationships relevant to the contents of this paper to disclose.
