# Supplementary material for: Artificial Intelligence–Driven Electrocardiogram Screening for Asymptomatic Left Ventricular Systolic Dysfunction in the General Population
Source: JACC Adv. 2026 Mar 18;5(4):102660. doi: 10.1016/j.jacadv.2026.102660 (PMC13015574; doi:10.1016/j.jacadv.2026.102660)
Supplement: Supplementary data [file mmc1.docx]

**Supplementary Marterials**

**Supplementary method 1.** AI-ECG Model Architecture and Training

**Supplementary figure 1.** Distribution of Risk Scores

**Supplementary figure 2.** Longitudinal AiTiALVSD Scores in True-Positive LVSD Patients Prior to Diagnosis

**Supplementary Table 1** Confusion Matrix of AiTiALVSD Classification

**Supplementary Table 2.** Diagnostic Performance of the AiTiALVSD Model in the Index ECG–TTE Pair Analysis (Patient-Level Sensitivity Analysis)

**Supplementary Table 3.** Diagnostic Performance of the AiTiALVSD Model Across Risk Score Thresholds (LVEF <40%)

**Supplementary Table 4.** Diagnostic Performance of the AiTiALVSD Model Across Risk Score Thresholds (LVEF <50%)

**Supplementary method 1.** AI-ECG Model Architecture and Training

**AI-ECG Model Architecture and Training**

The AI-enabled electrocardiogram (AI-ECG) model evaluated in this study, AiTiALVSD version 1.00.00 (Medical AI Co., Ltd.), was developed using a deep learning framework based on a residual neural network (ResNet) architecture to analyze standard 12-lead ECG signals for the detection of left ventricular systolic dysfunction (LVSD). The network begins with an initial one-dimensional convolutional block, followed by four sequential feature extraction blocks, each consisting of three residual blocks, and concludes with a fully connected output layer.

Within each residual block, the network comprises one-dimensional convolutional layers, batch normalization, rectified linear unit (ReLU) activation functions, and dropout layers. Skip connections were incorporated to facilitate stable gradient propagation and deep feature learning. This architectural design enables effective extraction of temporal and morphological features from raw ECG waveforms.

**Training Strategy and Loss Functions**

The model was trained using a multi-task learning framework designed to simultaneously perform binary classification for LVSD detection and regression to estimate continuous left ventricular ejection fraction (LVEF) values. This dual-objective approach allowed the model to capture both categorical and continuous representations of systolic dysfunction using ECG signals alone, without the inclusion of additional clinical variables.

For the classification task, cross-entropy loss was employed, whereas mean squared error loss was used for the regression task. The overall training objective optimized a weighted combination of these loss functions to improve predictive performance and model stability.

**Training Dataset and Optimization**

Model training was performed on a large dataset comprising 364,845 digital 12-lead ECG recordings collected from four South Korean hospitals. The dataset was randomly partitioned into training (80%), validation (15%), and testing (5%) subsets, with strict separation at the patient level to ensure that no individual appeared in more than one subset.

Optimization was conducted using the Adam optimizer with an initial learning rate of 0.0001. A cosine warm-up learning rate scheduler was applied to improve convergence during training. The model was trained for up to 150 epochs using a population-based training strategy, with hyperparameters optimized based on performance on the validation set.

**Model Selection and Output**

Final model selection was guided by multiple performance metrics, including the area under the receiver operating characteristic curve (AUROC), the area under the precision–recall curve (AUPRC), overall loss, and F1 score. The trained model outputs a continuous probability score reflecting the risk of LVSD, reported on a scale from 0 to 100 with a precision of one decimal place.

**Supplementary figure 1.** Distribution of Risk Scores


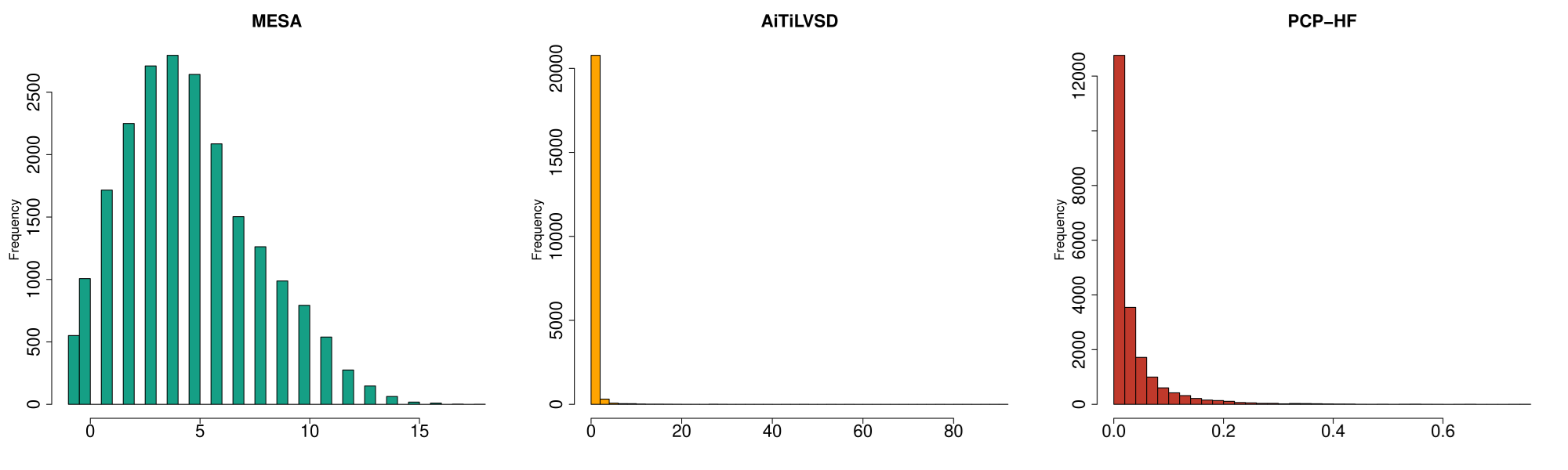


Histograms of risk score distributions for three models in the study population (n = 21,357). The left panel shows the distribution of the MESA 5-year heart failure (HF) risk scores, the middle panel depicts the AiTiALVSD probability scores, and the right panel presents the PCP-HF scores. Each model demonstrates a distinct distribution pattern, with the AiTiALVSD scores exhibiting a highly right-skewed pattern, reflecting the low prevalence of asymptomatic LVSD in the screening cohort.

MESA, Multi-Ethnic Study of Atherosclerosis; PCP-HF, Pooled Cohort Equations to Prevent HF.

**Supplementary figure 2.** Longitudinal AiTiALVSD Scores in True-Positive LVSD Patients Prior to Diagnosis


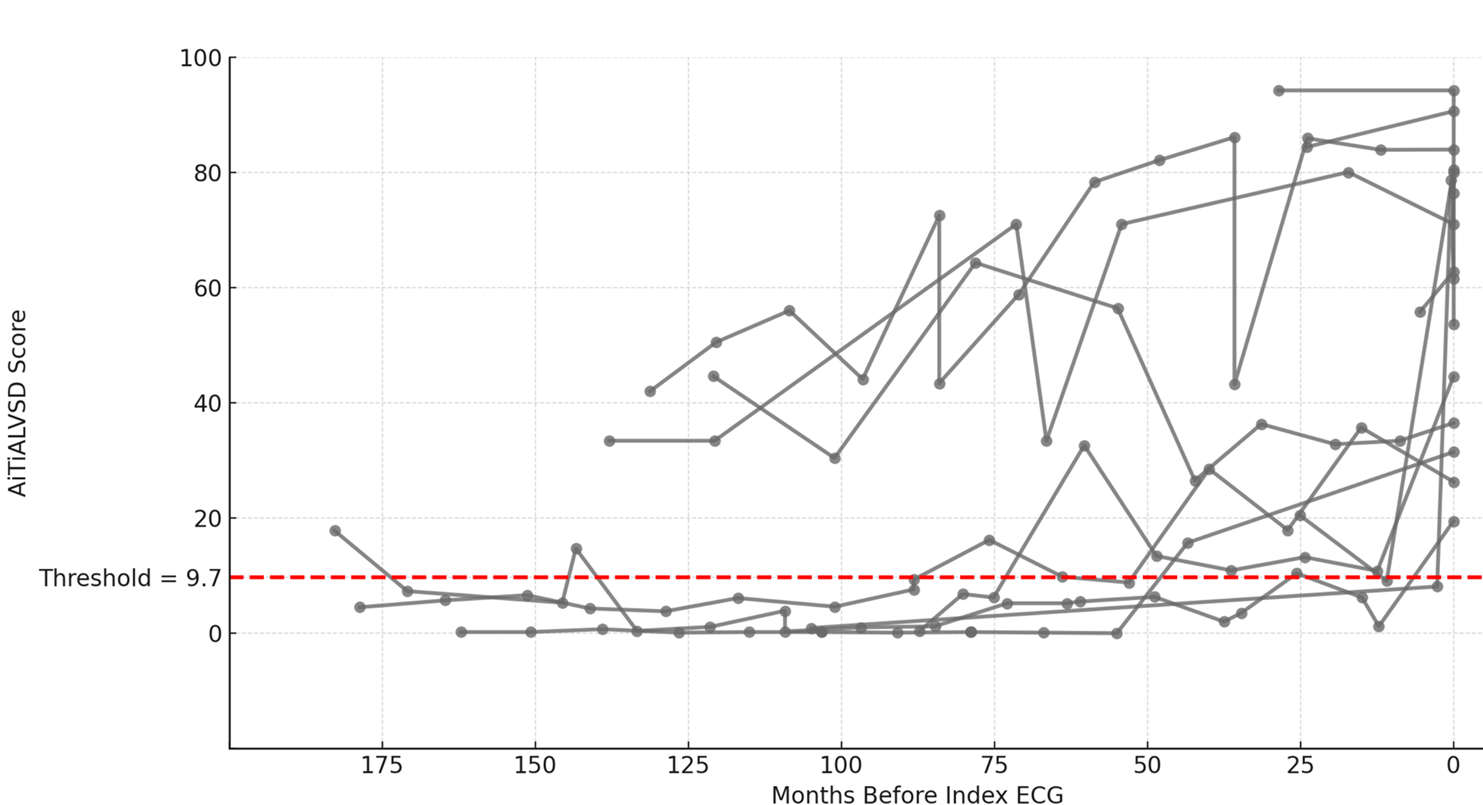


Trajectory of AiTiALVSD scores over time among 12 patients with confirmed asymptomatic LVSD (LVEF ≤40%) who had at least one prior ECG before the index echocardiogram. The x-axis represents months before the index ECG date, and the y-axis represents the AiTiALVSD score. The red dashed line indicates the pre-specified diagnostic threshold of 9.7. Notably, 12 of the 16 patients exceeded the threshold in their earlier ECGs, indicating a potential missed opportunity for earlier detection.

LVSD, left ventricular systolic dysfunction; LVEF, left ventricular ejection fraction; ECG, electrocardiogram.

**Supplementary table 1.** Confusion matrix of AiTiALVSD classification

|  | High risk | Low risk | Total |
| --- | --- | --- | --- |
| LVSD | 29 | 3 | 32 |
| Non-LVSD | 348 | 60,331 | 60,679 |
| Total | 377 | 60,334 | 60,711 |

In the AiTiALVSD model, the threshold for distinguishing between low and high risk of LVSD was determined based on the development set to ensure a sensitivity of 90%.

LVSD, left ventricular systolic dysfunction

**Supplementary Table 2.** Diagnostic Performance of the AiTiALVSD Model in the Index ECG–TTE Pair Analysis (Patient-Level Sensitivity Analysis)

|  | **AUROC (95% CI)** | **AUPRC (95% CI)** | **Sensitivity (95% CI)** | **Specificity (95% CI)** | **PPV (95% CI)** | **NPV (95% CI)** |
| --- | --- | --- | --- | --- | --- | --- |
| **LVEF ≤40%** | 0.962 (0.879–0.999) | 0.310 (0.156–0.505) | 0.869 (0.722–1.000) | 0.995 (0.994–0.996) | 0.089 (0.054–0.126) | 1.000 (1.000–1.000) |
| **LVEF ≤50%** | 0.925 (0.883–0.961) | 0.353 (0.246–0.466) | 0.575 (0.474–0.677) | 0.996 (0.995–0.997) | 0.254 (0.198–0.310) | 0.999 (0.999–0.999) |

This table presents diagnostic performance metrics of the AiTiALVSD model based on one index ECG–TTE pair per individual (n = 40,713), defined as the most recent eligible ECG–TTE encounter for each participant.

TTE, transthoracic echocardiography; LVEF, left ventricular ejection fraction; AUROC, area under the receiver operating characteristic curve; AUPRC, area under the precision–recall curve; PPV, positive predictive value; NPV, negative predictive value; CI, confidence interval

**Supplementary Table 3.** Diagnostic Performance of the AiTiALVSD Model Across Risk Score Thresholds (LVEF <40%)

| **AiTiALVSD Threshold** | **Sensitivity (%)** | **Specificity (%)** | **PPV (%)** | **NPV (%)** | **TP** | **FP** | **FN** | **TN** |
| --- | --- | --- | --- | --- | --- | --- | --- | --- |
| 9.7 (current cutoff) | 90.6 | 99.4 | 7.7 | 100 | 29 | 348 | 3 | 60331 |
| 10 | 90.6 | 99.4 | 8 | 100 | 29 | 335 | 3 | 60344 |
| 15 | 87.5 | 99.6 | 11.4 | 100 | 28 | 218 | 4 | 60461 |
| 20 | 84.4 | 99.7 | 14.2 | 100 | 27 | 163 | 5 | 60516 |
| 25 | 84.4 | 99.8 | 16.9 | 100 | 27 | 133 | 5 | 60546 |
| 30 | 78.1 | 99.8 | 20.8 | 100 | 25 | 95 | 7 | 60584 |
| 35 | 75 | 99.9 | 24.7 | 100 | 24 | 73 | 8 | 60606 |
| 40 | 65.6 | 99.9 | 26.2 | 100 | 21 | 59 | 11 | 60620 |

PPV, positive predictive value; NPV, negative predictive value; TP, true positive; FP, false positive; FN, false negative; TN, true negative.

**Supplementary Table 4.** Diagnostic Performance of the AiTiALVSD Model Across Risk Score Thresholds (LVEF <50%)

| **AiTiALVSD Threshold** | **Sensitivity (%)** | **Specificity (%)** | **PPV (%)** | **NPV (%)** | **TP** | **FP** | **FN** | **TN** |
| --- | --- | --- | --- | --- | --- | --- | --- | --- |
| 9.7 (current cutoff) | 58.6 | 99.5 | 24.9 | 99.9 | 94 | 283 | 66 | 60268 |
| 10 | 58.8 | 99.6 | 25.8 | 99.9 | 94 | 270 | 66 | 60281 |
| 15 | 50.6 | 99.7 | 32.9 | 99.9 | 81 | 165 | 79 | 60386 |
| 20 | 45 | 99.8 | 37.9 | 99.9 | 72 | 118 | 88 | 60433 |
| 25 | 44.4 | 99.9 | 44.4 | 99.9 | 71 | 89 | 89 | 60462 |
| 30 | 38.1 | 99.9 | 50.8 | 99.8 | 61 | 59 | 99 | 60492 |
| 35 | 33.8 | 99.9 | 55.7 | 99.8 | 54 | 43 | 106 | 60508 |
| 40 | 31.2 | 100 | 62.5 | 99.8 | 50 | 30 | 110 | 60521 |

PPV, positive predictive value; NPV, negative predictive value; TP, true positive; FP, false positive; FN, false negative; TN, true negative.
